# Identification and Validation of a Novel Six-lncRNA-Based Prognostic Model for Lung Adenocarcinoma

**DOI:** 10.3389/fonc.2021.775583

**Published:** 2022-01-17

**Authors:** Lingge Yang, Yuan Wu, Huan Xu, Jingnan Zhang, Xinjie Zheng, Long Zhang, Yongfang Wang, Weiyu Chen, Kai Wang

**Affiliations:** ^1^ Department of Respiratory Medicine, The Fourth Affiliated Hospital, College of Medicine, Zhejiang University, Yiwu, China; ^2^ Department of Respiratory Medicine, The Second Affiliated Hospital, Zhejiang University School of Medicine, Hangzhou, China

**Keywords:** lung adenocarcinoma, lncRNA, prognostic model, least absolute shrinkage and selection operator (LASSO), overall survival

## Abstract

**Objective:**

This study was conducted in order to establish a long non-coding RNA (lncRNA)-based model for predicting overall survival (OS) in patients with lung adenocarcinoma (LUAD).

**Methods:**

Original RNA-seq data of LUAD samples were extracted from The Cancer Genome Atlas (TCGA) database. Univariate Cox survival analysis was performed to select lncRNAs associated with OS. The least absolute shrinkage and selection operator (LASSO) regression analysis and multivariate Cox analysis were performed for building an OS-associated lncRNA prognostic model. Moreover, receiver operating characteristic (ROC) curves were generated to assess predictive values of the hub lncRNAs. Consequently, qRT-PCR was conducted to validate its prognostic value. The potential roles of these lncRNAs in immunotherapy and anti-angiogenic therapy were also investigated.

**Results:**

The lncRNA-associated risk score of OS (LARSO) was established based on the LASSO coefficient of six individual lncRNAs, including CTD-2124B20.2, CTD-2168K21.1, DEPDC1-AS1, RP1-290I10.3, RP11-454K7.3, and RP11-95M5.1. Kaplan–Meier analysis revealed that LUAD patients with higher LARSO values had a shorter OS. Furthermore, a new risk score (NRS), including LARSO, stage, and N stage, could better predict the prognosis of LUAD patients compared with LARSO alone. Evaluation of the prognostic model in our cohort demonstrated that patients with higher scores had a worse prognosis. In addition, correlation analysis between these six lncRNAs and immune checkpoints or anti-angiogenic targets suggested that LUAD patients with high LARSO might not be sensitive to immunotherapy or anti-angiogenic therapy.

**Conclusions:**

This robust six-lncRNA prognostic signature may be used as a novel and powerful prognostic biomarker for lung adenocarcinoma.

## Introduction

Lung cancer is the leading cause of cancer-related morbidity and mortality worldwide (1). Lung adenocarcinoma (LUAD), the most common histological type of lung cancer, is highly heterogeneous and accounts for approximately 40% of all lung cancer cases (2). Although advances have been made in improving diagnosis and developing new treatments, the overall survival of (OS) patients has not significantly improved, with 5-year survival rates being <18%. One of the main reasons for poor prognosis is that most patients are diagnosed only when in an advanced stage, thus losing the chance to undergo surgery (3). Therefore, identifying accurate prognostic biomarkers for early lung cancer diagnosis, especially for LUAD, remains of crucial importance.

Long non-coding RNAs (lncRNAs) are widely defined as RNA transcripts lacking protein-coding abilities, with a length longer than 200 nucleotides (4, 5). lncRNAs are essential in the regulation of various cellular and physiologic functions, including gene activation/silencing (6, 7), chromatin dynamic (8), post-translational modification (9), and alternative splicing (10), and have been reported to be involved in tumorigenesis and tumor metastasis (11, 12). For example, lncRNAs named HOTAIR could serve as a modular scaffold to reprogram chromatin state, promoting cancer metastasis (13). Moreover, DLX6AS lncRNA acts as competing endogenous RNAs (ceRNAs) that can sponge target microRNAs or proteins and can promote cancer proliferation and invasion by reducing the endogenous function of miR-181b in pancreatic cancer (14, 15). Human colorectal cancer-specific CCAT1 lncRNA can inhibit long-range chromatin interactions with its enhancers (16).

Increasing evidence suggests that aberrant expression of lncRNAs is associated with various human cancers, such as ovarian (17) and non-small cell lung cancer (NSCLC) (18). Notably, some lncRNAs have been implicated as effective biomarkers for cancer diagnosis and prognostication (19). In this study, we aimed to identify and validate potential lncRNA biomarkers for the diagnosis and prognosis of LUAD. RNA sequencing (RNA-seq) data for LUAD were retrieved from The Cancer Genome Atlas (TCGA). A novel lncRNA-based prognostic signature was discovered for LUAD based on bioinformatics approaches. The six-lncRNA signature illustrated desirable sensitivity and specificity after collecting validation sets and follow-up.

## Material and Methods

### Data Collection and Processing

HTseq-FPKM (fragments per kilobase million), the VarScan2 data of exon group mutation, and clinical information of LUAD patients were downloaded from TCGA (https://portal.gdc.cancer.gov) (20). FPKM was transferred into TPM (transcripts per million). In addition, the expression matrix of lncRNAs was extracted.

### Univariate Cox Survival Analysis

The LUAD patients were grouped based on the median value of lncRNA expression. Based on clinical information of LUAD patients, univariate survival analysis of overall survival (OS) was performed by survival package (21) of R 4.0.2 software. The lncRNAs associated with OS were significantly extracted for further model building.

### OS-Associated Prognostic Model Building

lncRNA expression matrix associated with OS as well as clinical information of related TCGA-LUAD patients was retrieved. The least absolute shrinkage and selection operator (LASSO) regression model was built according to the survival state of patients (i.e., dead or alive) by glmnet package (nfold = 10, *λ* = lambda.min) (22). The lncRNAs whose regression coefficients were not 0 were included for multivariate Cox regression analysis, and forestplot package (23) was used for analyzing and plotting. The risk score equation named lncRNA-associated risk score of OS (LARSO) was obtained depending on the coefficients of lncRNAs associated with prognosis in the regression model. The ggplot2 package (24) and timeROC package (25) were used to generate a scatter map and a heat map of lncRNA expression after calculating the LARSO value of each sample. Time-dependent (1, 3, and 5 years) receiver operating characteristic (ROC) and Kaplan–Meier curves were also generated. The largest Youden index of ROC was selected as the best cutoff value of LARSO, and the patients were divided into high-risk and low-risk groups according to this value. Nomogram and calibrate curves were plotted using an rms package (26). The results of the Cox regression analysis were visualized.

### Correlation Analysis Between LARSO and Clinicopathological Features of TCGA-LUAD

Clinical information and the key gene (EGFR, KRAS, ALK, ROS1, and BRAF) mutation status of LUAD patients in TCGA were extracted for investigating the correlation between LARSO and clinicopathological features. Univariate Cox regression analysis was performed to pick out factors correlated with OS, and further multivariate Cox regression analysis was performed to obtain independent prognosis factors associated with OS. Furthermore, we analyzed the correlation between LARSO and these factors overall and the differences between high- and low-risk groups. In order to obtain a new risk score (NRS), LASSO regression analysis was carried out again according to the method described above. Prognostic values of the novel prognostic model with clinical characteristics were reanalyzed.

### Validation Set Collection and Follow-Up

LUAD tissues and paired normal adjacent tissues were collected from 48 LUAD patients in the Second Affiliated Hospital of Zhejiang University School of Medicine from March 2018 to August 2020 (Approval No. IR2019001101; approval on April 3, 2019). Clinicopathological characteristics and prognostic survival information included age, sex, smoking habit, tumor size, pathogenic site, and clinical TNM stage. The follow-up date ended on June 8 in 2021, and outpatient and telephone follow-up were performed.

This study was approved by the Institutional Review Committee of the Second Affiliated Hospital of Zhejiang University School of Medicine. All the patients and their guardians gave written informed consent before surgery.

### qRT-PCR

Total RNA in the tumor samples was extracted using the RNA-Quick Purification Kit (RN001, ES Science, Beijing, China) according to the instructions of the manufacturer. One microgram of RNA was then reverse-transcribed into cDNAs using the PrimeScript™ RT Reagent Kit (RR037A, TaKaRa, Japan). qRT-PCR analysis was performed using TB Green Premix Ex Taq™ kit (RR420A, TaKaRa, Japan) on a CFX96 Real-Time System (Bio-Rad, UK). β-Actin was used as the housekeeping gene, and the primer information is listed in **Table S1**.

### Validation of Prognostic Value

The CT values of the first LUAD patient sample for qPCR were set as control. 2^−△△CT^ was performed for all the samples to obtain the relative expression values of lncRNAs in the prognostic model. Relative LARSO and NRS values of LUAD patients were calculated by the risk score equation. These results were integrated with the prognosis information of patients. Hence, the related scatter plot, heat map, ROC curve, and Kaplan–Meier curve were analyzed and plotted to validate the prognostic value of our model.

### Gene Set Enrichment Analysis

The patients were divided into high-risk and low-risk groups according to the cutoff value of LARSO in the modeling set. KEGG and hallmark pathway enrichment analyses in gene set enrichment analysis (GSEA, https://www.gsea-msigdb.org/gsea) were performed for TCGA transcriptome matrix normalized by TPM. The results (FDR < 0.05) were visualized (27).

### Correlation Analysis Between Immune-Related Genes and lncRNAs in the LARSO Prognostic Model

Immune-related genes (IRGs) were downloaded from the ImmPort database (https://immport.niaid.nih.gov) (28). IRG expression matrix profiles, including antigen processing and presentation, interferons and interferon receptors, TCR signaling pathway, TNF family members and receptors, and TGFb family members and receptors, were extracted from the TCGA transcriptome matrix profile described above. The correlation coefficient between IRGs and lncRNA in the prognostic model was calculated by the psych package (29) and plotted by the ggplot2 package (24).

### Correlation Analysis of Immune Infiltration

Score evaluation of 22 immune cells in the TCGA transcriptome matrix profile described above was performed in the CIBERSORTx database (https://cibersort.stanford.edu/) (30, 31). The correlation between these scores and lncRNA expression was analyzed. Besides, differences in immune cell scores between high- and low-risk groups were analyzed to evaluate the correlation between the prognostic model and immune infiltration.

### Correlation Analysis of Immune Checkpoints and Anti-Angiogenic Targets With lncRNAs in the LARSO Prognostic Model

Expression matrices of immune checkpoints including PDCD1 (PD-1), CD274 (PD-L1), PDCD1LG2 (PD-L2), and CTLA4 were extracted from the transcriptome matrix in TCGA described above. Expression matrices of anti-angiogenic targets including KDR (VEGFR-2), FLT4 (VEGFR-3), FLT1 (VEGFR-1), EGFR, PDGFRB (PDGFR-2), KIT, PDGFRA (PDGFR-1), and FGFR1-4 were also extracted from this matrix in TCGA. The correlation between these immune checkpoints or targets and lncRNA expression was analyzed and plotted. We also compared the differences of these immune checkpoints or targets between the high- and low-risk groups.

### Statistical Analysis

The statistical results were analyzed and plotted by R 4.0.2 and GraphPad 8.0 software. Mean ± standard deviation (SD) displayed the measurement data. Student’s *t*-test was used to compare the difference between high- and low-risk groups. A chi-square test was used to compare the difference between high- and low-risk groups for enumeration data. Spearman test was used for correlation analysis, and log-rank tests were used for survival analysis. *P <*0.05 was considered statistically significant (*P < 0.05, **P < 0.01, ***P < 0.001, ****P < 0.0001).

## Results

### Construction of a Regression Equation Based on the Six-lncRNA Prognostic Risk Scoring Model

**Figure 1:**
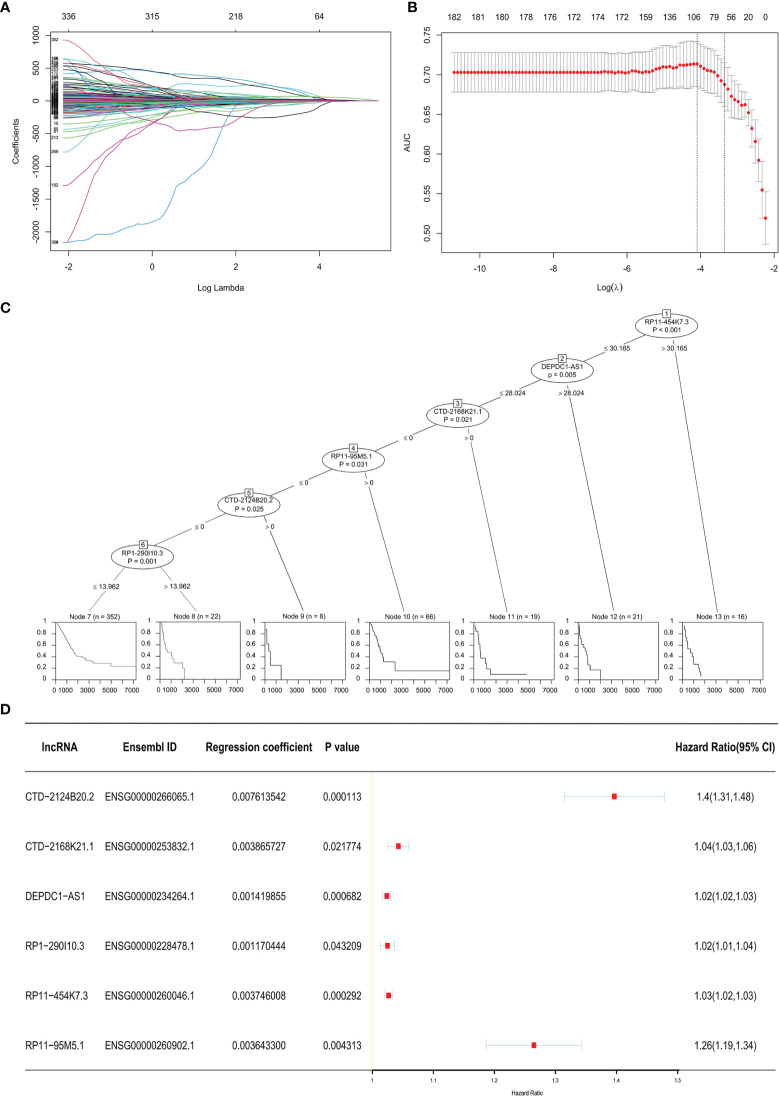
Prognostic model. **(A)** Least absolute shrinkage and selection operator (LASSO) coefficient distribution diagram of 336 long non-coding RNAs (lncRNAs): *x*-coordinate was log (*λ*) for screening the best tuning parameter (*λ*). **(B)** Tuning parameter (*λ*) in the LASSO regression model was selected according to 10-fold cross-validation. Plotting was performed based on this value as well as the area under the receiver operating characteristic curve. A vertical dashed line was drawn at the best value by using the minimum standard and 1 standard error of the minimum standard (1-SE standard). **(C)** Visualization of the Cox regression analysis: the significant lncRNAs (*P* < 0.05) in the Cox regression were displayed. The impacts of these lncRNAs on the prognosis of lung adenocarcinoma (LUAD) patients are shown below by a single survival curve. **(D)** Forest map with HR: the Ensembl ID of the six lncRNAs, regression coefficient in LASSO regression, and *P*-value in the Cox regression analysis were displayed; a vertical line was drawn at HR = 1.

Three hundred thirty-six lncRNAs correlated with OS were retrieved from the TCGA database. There were only 20 lncRNAs that exhibited effective co-efficiency in regression *via* LASSO regression analysis (**Figures 1A, B**). Among them, six lncRNAs, i.e., CTD-2124B20.2, CTD-2168K21.1, DEPDC1-AS1, RP1-290I10.3, RP11-454K7.3, and RP11-95M5.1, were finally identified *via* expression profile analysis as well as Cox regression analysis. All six lncRNAs showed correlations with the status of the patients (alive or dead) and significantly affected OS (**Figure 1C**). Based on the results above, we obtained a LARSO regression equation: LARSO = 0.007613542 × CTD-2124B20.2 expression value + 0.003865727 × CTD-2168K21.1 expression value + 0.001419855 × DEPDC1-AS1 expression value + 0.001170444 × RP1-290I10.3 expression value + 0.003746008 × RP11-454K7.3 expression value + 0.0036433 × RP11-95M5.1 expression value. In addition, correlation coefficients of the six lncRNAs were >0 in the regression equation, which implied that these lncRNAs were potential oncogenic factors. Next, hazard ratio (HR) values further confirmed that lncRNAs were potential oncogenes (HR > 1). Hence, it was suggested that the selected six lncRNAs were risk factors of LUAD (**Figure 1D**).

### Construction of a Six-lncRNA Signature for Predicting OS

**Figure 2:**
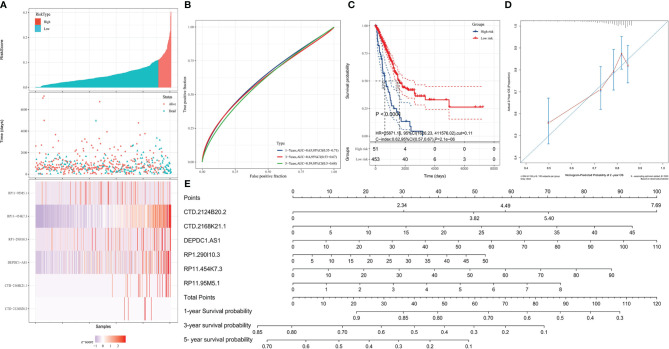
Validation of the prognostic model. High- and low-risk groups were divided according to the lncRNA-associated risk score of OS (LARSO) value. **(A)** The histogram, scatter plot, and heat map showed the risk grouping, patient survival status, and the expression of the six lncRNAs, respectively. **(B)** Receiver operating characteristic (ROC) curves were shown at 1, 3, and 5 years based on the LARSO scores and prognosis of LUAD patients. **(C)** Kaplan–Meier survival curves were plotted: the overall survival time of patients in the high-risk group was significantly shorter than that in the low-risk group (*P* < 0.0001). **(D)** Calibration curve: the *x*-coordinate was the probability of 2-year survival predicted by the model, and the *y*-coordinate represented the actual 2-year survival. **(E)** Column map: the 1-, 3-, and 5-year survival rates of patients could be predicted by scoring the expression level of the six lncRNAs. A log-rank test was used for survival analysis.

Based on the LARSO regression equation above, LARSO values of each LUAD case in the TCGA database were calculated. Afterwards, all the TCGA-LUAD patients were divided into high-risk (*n* = 48) and low-risk groups (*n* = 465) according to the cutoff point (LARSO = 0.110). Next, we analyzed the lncRNA relative expression of every TCGA-LUAD patient, the expression distribution of lncRNAs, and the survival state of patients, both in the high- and low-risk groups (**Figure 2A**). The relative expression of six lncRNAs and the number of deaths were higher in the high-risk group compared with those in a low-risk group. Notably, the six-lncRNA signature reached AUC values of 0.63 in the 1-year ROC curve, 0.6 in the 3-year ROC curve, and 0.59 in the 5-year ROC curve, suggesting an effective performance in OS prediction (**Figure 2B**). Moreover, Kaplan–Meier curves (**Figure 2C**) suggested statistically significant differences between the high- and low-risk groups (*P* < 0.0001). Similarly, a significant difference was also observed in median survival time between the high-risk group (624 days) and the low-risk group (1,559 days).

By analyzing the prediction accuracy through calibration curves, we demonstrated that the 2-year OS prediction had the highest accuracy *via* the six-lncRNA signature prognostic model (**Figure 2D**). A nomogram was also built to evaluate the prediction abilities of this prognostic model on 1-, 3-, and 5-year survival probability (**Figure 2E**). This result suggested that the expression of these six lncRNAs was negatively correlated with patient survival, i.e., lower expression, higher survival.

### LARSO Was Correlated With Clinicopathologic Features of LUAD Patients

The correlation analysis between LARSO and the clinicopathologic features of TCGA-LUAD was then investigated. As shown in **Table 1**, statistical differences between the high- and low-risk groups were found in T stage (*P* < 0.0001), N stage (*P* = 0.0342), survival status (*P* < 0.0001), and cancer status (*P* = 0.0002). Moreover, higher LARSO values implied larger tumor size, lymph nodes metastasis, more death of LUAD patients, or more survival of patients with tumor.

**Table 1:** Correlation between LARSO and the clinicopathological characteristics of lung adenocarcinoma patients in the TCGA database (*N* = 514).

Variables	Low risk (*N* = 465)	High risk (*N* = 48)	*χ* ^2^	*t*	*P*-value
**Gender**					
Female	252	24	0.3078		0.5790
Male	213	24			
**Age (years)**	65.22 ± 9.94	66.06 ± 11.00		0.5486	0.5835
**Histological type**					
Lung acinar adenocarcinoma	18	0	7.045		0.7954
Lung adenocarcinoma mixed subtype	93	13			
Lung adenocarcinoma—not otherwise specified (NOS)	289	31			
Lung bronchioloalveolar carcinoma mucinous	5	0			
Lung bronchioloalveolar carcinoma non-mucinous	19	0			
Lung clear cell adenocarcinoma	1	0			
Lung micropapillary adenocarcinoma	3	0			
Lung mucinous adenocarcinoma	2	0			
Lung papillary adenocarcinoma	20	3			
Lung signet ring adenocarcinoma	1	0			
Lung solid pattern predominant adenocarcinoma	5	0			
Mucinous (colloid) carcinoma	9	1			
**Clinical stage**					
Unknown	8	0			
Stage I	253	21	5.221		0.1563
Stage II	110	11			
Stage III	73	11			
Stage IV	21	5			
**T classification**					
Tx	3	0			
T1	156	12	25.04		<0.0001****
T2	252	24			
T3	43	4			
T4	11	8			
Unknown	0	0			
**N classification**					
Nx	11	0			
N0	305	25	4.487		0.0342*
N1/N2/N3	148	23			
Unknown	1	0			
**M classification**					
Mx	132	8			
M0	309	35	1.422		0.2330
M1	20	5			
Unknown	4	0			
**Anatomic neoplasm subdivision**					
L-lower	69	8	0.9692		0.9144
L-upper	110	12			
R-lower	89	7			
R-middle	19	2			
R-upper	168	14			
Discrepancy/unknown	10	5			
**ECOG performance status**					
0	93	7	2.066		0.5588
1	100	14			
2	21	2			
3	3	0			
Unknown	296	25			
**Drug therapy**					
No	304	32	0.032		0.8579
Yes	161	16			
**Drug response (at the last time)**					
Yes (complete response + partial response + stable disease)	75	3	1.049		0.3058
No (progressive disease)	22	3			
Unknown	64	10			
**Radiotherapy**					
No	379	35	2.061		0.1511
Yes	86	13			
**Radiotherapy response (at the last time)**					
Yes (complete response + partial response + stable disease)	24	0	3.214		0.0730
No (progressive disease)	21	5			
Unknown	41	8			
**EGFR mutation**					
No	403	45	1.385		0.2393
Yes	62	3			
**KRAS mutation**					
No	334	40	2.362		0.1243
Yes	131	8			
**ALK mutation**					
No	431	45	0.0005		0.9822
Yes	34	3			
**ROS1 mutation**					
No	444	43	2.041		0.1531
Yes	21	5			
**BRAF mutation**					
No	427	45	0.035		0.8509
Yes	38	3			
**Follow-up**					
Alive	313	8	48.82		<0.0001****
Dead	144	39			
Unknown	8	1			
**Cancer status**					
Tumor free	286	20	14.36		0.0002***
With tumor	89	21			
Discrepancy/unknown	90	7			

Patients were divided into the high-risk group (n = 48) and low-risk group (n = 465) according to LASSO = 0.110. Bold values indicate P < 0.05 and *P < 0.05, ***P < 0.001, and ****P < 0.0001.

**Figure 3:**
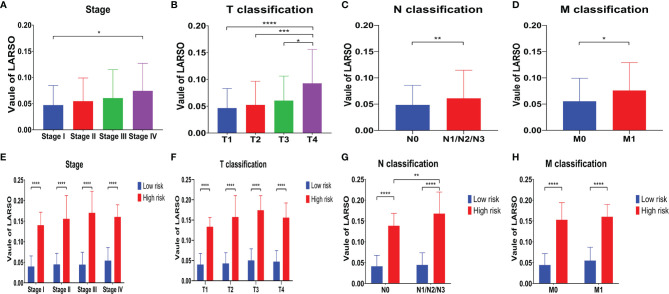
Correlation analysis between the LARSO value and stage or TNM stage. **(A)** Tumor stage was negatively correlated with LARSO value, i.e., higher stage, lower LARSO value; the difference of LARSO between stage I and stage IV was statistically significant (*P* < 0.05). **(B)** A higher LARSO value was associated with a worse T stage. The LARSO value at T1, T2, or T3 was statistically different from the LARSO value at T4. **(C)** The LARSO value of patients with lymph node metastasis (N1/N2/N3) was significantly higher than patients without lymph node metastasis (N0). **(D)** LARSO value in the M0 group was significantly lower than that in the M1 group (*P* < 0.05). **(E)** The LARSO value between the high- and low-risk groups at different stages was statistically significant, and the LARSO value of the high-risk group was significantly higher than that of the low-risk group (*P* < 0.05). **(F)** The LARSO value in different T stages between the high- and low-risk groups was statistically significant. **(G)** The LARSO value between the high- and low-risk groups was significantly different in various N stages (*P* < 0.05). In addition, the LARSO value in the high-risk group with lymph node metastasis (N1/N2/N3) was significantly higher than that in the non-lymph node metastasis group (N0). **(H)** The difference of LARSO value in different M stages in the high- and low-risk groups was statistically significant. Student’s *t*-test was used for testing between groups. *P < 0.05; **P < 0.01; ***P < 0.001; ****P < 0.0001.

Furthermore, we conducted univariate and multivariate Cox regression analysis for the above factors (**Table 2**). Group (HR = 0.3519, *P* < 0.001), LARSO (HR = 26401, *P* < 0.001), stage, and TNM stage were all correlated with OS. Above all, LARSO (HR = 1,867.458, *P* = 0.00642) and T3 stage (HR = 2.644, *P* = 0.00963) were two independent prognostic factors affecting OS. Thus, we analyzed the correlation between LARSO value and stage or TNM stage and investigated whether there was a significant difference in LARSO value between high- and low-risk groups at a different stage or TNM stage. Our data indicated that a higher LARSO value was correlated with worse or severe TNM stage. Moreover, there were significant differences in LARSO value between stage I and stage IV (*P* = 0.0125, **Figure 3A**), or T1 and T4 (*P* < 0.0001), or T2 and T4 (*P* = 0.0005), or T3 and T4 (*P* = 0.0288, **Figure 3B**). Also, the LARSO value was different in the N0 and N1/N2/N3 groups (*P* = 0.0070, **Figure 3C**) and in the M0 and M1 groups (*P* = 0.0262, **Figure 3D**).

**Table 2:** Univariate and multivariate analyses for factors influencing overall survival (OS) of lung adenocarcinoma patients in the TCGA database.

Variables		Overall survival (OS)			
		Univariate		Multivariate	
		HR (95% CI)	*P*-value	HR (95% CI)	*P*-value
Group (low risk)		0.3519 (0.2467–0.5019)	<0.001***	–	–
LARSO		26,401 (1,640–424,906)	<0.001***	1,867.458 (8.299–42,020)	0.00642**
Stage	Stage II	2.472 (1.718–3.557)	<0.001***	–	–
	Stage III	3.494 (2.383–5.124)	<0.001***	–	–
	Stage IV	3.817 (2.199–6.624)	<0.001***	–	–
T	T2	1.452 (1.017~2.073)	0.0398*	–	–
	T3	2.958 (1.758–4.979)	<0.001***	2.644 (1.266–5.515)	0.00963**
	T4	2.914 (1.501–5.659)	0.00159**	–	–
N (N0)		0.388 (0.288–0.521)	<0.001***	–	–
M (M1)		2.133 (1.245–3.654)	0.00583**	–	–

All results were calculated by the survival package of R 4.0.2 software.

TCGA, The Cancer Genome Atlas; CI, confidence interval; LARSO, lncRNA-associated risk score of overall survival. *P < 0.05, **P < 0.01, ***P < 0.001.

Next, we compared LARSO values in the high- and low-risk groups based on stage, T classification, N classification, or M classification. There were significant differences in LARSO value between the two groups on stage (*P* < 0.0001, **Figure 3E**) or T classification (*P* < 0.0001, **Figure 3F**). As for the N stage (**Figure 3G**), a significant difference in LARSO value was found between the high- and low-risk groups (*P* < 0.0001). Besides, there was a significant difference between N0 and N1/N2/N3 in the high-risk group (*P* = 0.0033). Likewise, the difference in LARSO value between the high- and low-risk groups in the M stage was also statistically significant (*P* < 0.0001, **Figure 3H**).

### LARSO Combined With Stages and N Stages Could Better Predict the Prognosis of LUAD Patients

Firstly, clinical factors correlated with OS were obtained *via* univariate Cox regression analysis. Then, these clinical factors were combined with the LARSO data, after which a LASSO regression analysis was performed. Therefore, a new risk score (NRS) was generated: 7.5671594 × LARSO + 0.3127315 × Stage score + 0.2828587 × N score. The scores of stages I, II, III, and IV were graded as 1, 2, 3, and 4, respectively. Similarly, the scores of NX (0), N0 (1), and N1/N2/N3 (2) were also defined.

**Figure 4:**
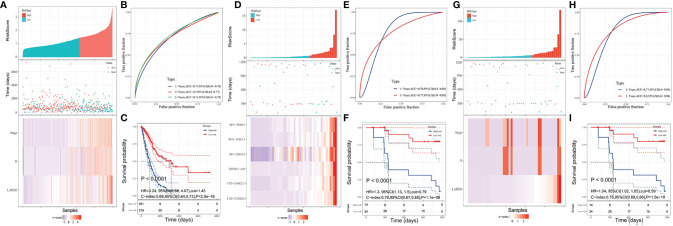
LARSO combined with stages and N stages for predicting the prognosis of LUAD patients. **(A)** Risk grouping situation, the scatter diagram of patient survival status, and the distribution of LARSO, stage, and N stage in each sample are shown. **(B)** The 1-, 3-, and 5-year ROC curves plotted based on NRS and patient survival. **(C)** Kaplan–Meier analysis: the overall survival time of patients in the high-risk group was significantly shorter than that in the low-risk group (*P* < 0.0001). **(D)** The LARSO values were calculated based on the survival state of 48 patients and the expression of the six lncRNAs. Histogram of the high- and low-risk distribution, scatter plot of patient survival, and the heat map of the expression of the six lncRNAs were displayed. **(E)** The 1- and 3-year ROC curves were drawn based on the LARSO value and the survival state of patients. **(F)** Kaplan–Meier analysis: the overall survival time of patients in the high-risk group was significantly shorter than that in the low-risk group (*P* < 0.0001). **(G)** The risk grouping, the scatter diagram of patient survival status, and the distribution of LARSO, stage, and N stage in each sample were shown after NRS was calculated. **(H)** According to the 1- and 3-year ROC curves drawn based on the NRS value and patient survival time, the AUC value of 1-year ROC was 0.77, while the AUC value of the 3-year ROC was up to 0.8. **(I)** Kaplan–Meier analysis: the overall survival time of patients in the high-risk group was significantly shorter than that in the low-risk group (*P* < 0.0001). A log-rank test was used for survival analysis.

Based on this NRS regression equation, we calculated the NRS values of each TCGA-LUAD patient. All the patients were divided into a high-risk group (*n* = 183) and a low-risk group (*n* = 317) based on the new cutoff point of the ROC curve (NRS = 1.45). Higher NRS scores and deaths were observed in the high-risk group compared with the low-risk group (**Figure 4A**). Moreover, we found that the AUC value was 0.71 in the 1-year ROC curve, 0.7 in the 3-year ROC curve, and 0.71 in the 5-year ROC curve, indicating NRS with better OS prediction ability compared with LARSO alone (**Figure 4B**), as well as stage (**Figure S1A**) or N stage (**Figure S1B**) alone.

Survival analysis was further performed, suggesting a significant difference in the median survival time, with 3,094 days in the low-risk group, which was about 3.58 times higher than the high-risk group (864 days) (*P* < 0.0001, **Figure 4C**). These results implied that LARSO combined with stage and N stage could support a desirable prediction for LUAD prognosis.

### LARSO and Derived NRS Prognostic Model Validation

To validate our six-lncRNA signature prognostic model, we collected 48 pairs of carcinoma tissues and normal adjacent tissues for investigating the relative expression of six lncRNAs *via* qRT-PCR. Then, the LARSO values of each LUAD patient were calculated through the LARSO regression model. Likewise, we divided all the LUAD patients into high-risk group (*n* = 15) and low-risk group (*n* = 33) according to a cutoff point in a 3-year ROC curve (LARSO = 0.790). The relative expression of these lncRNAs, expression distribution, and survival status of LUAD patients were also examined. As shown in **Figure 4D**, the relative expression of these six lncRNAs and deaths was lower in the low-risk group compared with those in the high-risk group. Besides, AUC values of 1-year and 3-year ROC curves were 0.78 and 0.77, which indicated that our LARSO prognostic model has a good prognostic value (**Figure 4E**). However, 5-year ROC curve was not available due to insufficient follow-up time. Survival curves were also performed. A low-risk group demonstrated a prolonged median survival time with 1,162 days follow-up deadline, which was significantly longer than that of the high-risk group (342 days) (*P* < 0.0001, **Figure 4F**).

Furthermore, NRS values in the validation set were calculated, and the cutoff value (NRS = 6.59) was used for dividing patients into high- (*n* = 15) and low-risk (*n* = 33) groups. Similar to previous data, the high-risk group was associated with higher scores and more deaths (**Figure 4G**). Although the AUC value in the 1-year ROC curve (AUC = 0.77) was close to the result in **Figure 4E** (0.78), the AUC value in the 3-year ROC curve (AUC = 0.8) was better than that in **Figure 4E** (0.77) (**Figure 4H**). In addition, Kaplan–Meier curves showed a significant difference between high- and low-risk groups, thus indicating that patients in the low-risk groups had better prognoses (*P* < 0.0001, **Figure 4I**).

### Related Hallmarks and Regulatory Pathways of lncRNAs in the LARSO Prognostic Model

Next, we performed GSEA analysis as well as KEGG pathway analysis, aiming to investigate the related hallmarks and regulatory pathways associated with lncRNAs. GSEA suggested that the six lncRNAs are involved in several pathways, including G2M checkpoint, mTORC1 signaling, E2F targets, MYC targets, DNA repair, glycolysis, oxidative phosphorylation, and reactive oxygen species pathway (*P* < 0.05 and FDR < 0.05, **Figure S2A**). Generally, these hallmark gene sets participate in cell cycle regulation, DNA damage repair, or tumor cell metabolism regulation. Besides, the six lncRNAs might also be involved in various pathways after GSEA analysis in the KEGG pathway, for instance, proteasome, cell cycle, and nucleotide excision repair (*P* < 0.05 and FDR < 0.05, **Figure S2B**). All GSEA results are shown in **Table S2**.

### Correlation Between Immunity and lncRNAs in the LARSO Prognostic Model

**Figure 5:**
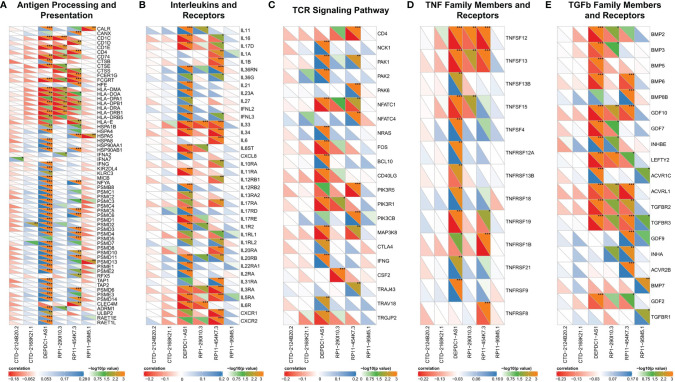
Correlated immune categories with lncRNAs in the prognostic model. **(A–E)** Correlation analysis between lncRNAs with gene expression in **(A)** antigen processing and presentation category, **(B)** interleukins and receptors category, **(C)** TCR signaling pathway category, **(D)** TNF family members and receptors category, and **(E)** TGFb family members and receptors category. The abscissa represents the lncRNAs, and the genes in the immune categories are on the ordinate. The lower left half triangle represents correlation: red means negative correlation, while blue implies positive correlation. Darker color means stronger correlation. The upper right semi-triangle is the value of −log10 (*P*-value). A larger value implies the darker the color. Spearman test was used for correlation analysis. *P < 0.05; **P < 0.01; ***P < 0.001.

In this part, we analyzed the correlation between immunity and lncRNAs in our prognostic model. We found that the correlation between lncRNAs and IRGs was different among six lncRNAs. Firstly, only DEPDC1-AS1 and RP11-454K7.3 had been found in the antigen processing and presentation category (**Figure 5A**). Most IRGs were associated with DEPDC1-AS1 or RP11-454K7.3 (*P* < 0.05), while merely a small part was correlated with RP1-290I10.3 or RP11-95M5.1 (*P* < 0.05). Also, CTD-2168K21.1 had no statistical significance with IRGs in this category. The analysis between these lncRNAs (except for RP11-95M5.1) and other categories, including interleukins and receptors (**Figure 5B**), TCR signaling pathway (**Figure 5C**), and TNF family members and receptors (**Figure 5D**), displayed similar results with antigen processing and presentation category.

RP11-95M5.1 showed no significant correlation with IRGs in these three categories. In addition, in the immune category of TGFb family members and receptors (**Figure 5E**), most IRGs were significantly correlated with DEPDC1-AS1, RP1-290I10.3, and RP11-454K7.3, while a small part was related to RP11-95M5.1. Hence, DEPDC1-AS1 and RP11-454K7.3 were more likely to regulate the above five immune categories, while RP1-290I10.3 and RP11-95M5.1 were less likely to be involved. CTD-2124B20.2 and CTD-2168K21.1 might not regulate the immune process of the above five categories.

### Immune Infiltration in the High- and Low-Risk Groups

**Figure 6:**
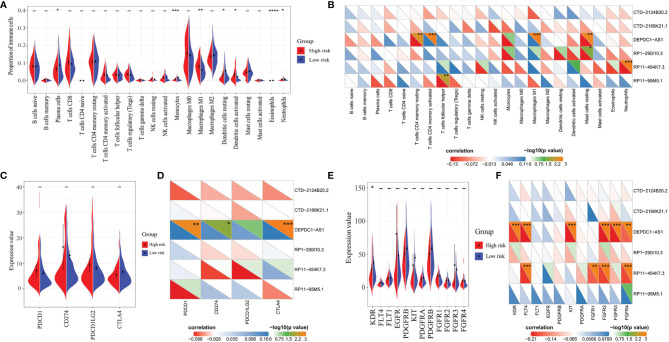
Correlation analysis of lncRNAs with immune cell infiltration score, immune checkpoint expression, or antivascular target expression between the high- and low-risk groups. **(A)** Significant immune infiltrates between the two groups: plasma cells (*P* = 0.0233), monocytes (*P* = 0.0003), macrophages M1 (*P* = 0.0013), dendritic cells resting (*P* = 0.0269), dendritic cells activated (*P* = 0.0294), eosinophils (*P* < 0.0001), and neutrophils (*P* = 0.0256). **(B)** Correlation analysis between lncRNAs in the prognostic model and immune infiltration score. **(C)** Correlation analysis of four immune checkpoints (PD-1, PD-L1, PD-L2, and CTLA4) between the high- and low-risk groups; no significant difference was found. **(D)** Correlation analysis between immune checkpoints with the six lncRNAs. **(E)** Correlation analysis of common anti-angiogenic targets between the high- and low-risk groups. Only KDR (VEGFR-2) showed a significant difference between the two groups (*P* = 0.0314). **(F)** Correlation analysis between anti-angiogenic targets with the six lncRNAs. Student’s *t*-test was used for the different tests between groups. Correlation analysis was performed by the Spearman test. *P < 0.05; **P < 0.01; ***P < 0.001; ****P < 0.0001.

Through immune infiltration analysis, different proportions of several immune cells were found between the high- and low-risk groups, including plasma cells (*P* = 0.0233), monocytes (*P* = 0.0003), macrophages M1 (*P* = 0.0013), dendritic cells resting (*P* = 0.0269), dendritic cells activated (*P* = 0.0294), eosinophils (*P* < 0.0001), and neutrophils (*P* = 0.0256) (**Figure 6A**). However, similar results were not observed in the correlation analysis between these immune cells and the six lncRNAs (**Figure 6B**). A positive correlation was only found between DEPDC1-AS1 and macrophages M1 (R = 0.276, *P* < 0.001), while a negative correlation was seen between RP11-454K7.3 and neutrophils (*R* = −0.118, *P* < 0.001). Therefore, the immune infiltration might slightly differ between the high- and low-risk groups. Moreover, the small difference of immune cells between the high- and low-risk groups, except for macrophages M1 and neutrophils, was more likely to result from the synergistic effect of all six lncRNAs in the LARSO prognostic model.

### Immunotherapy and Anti-Angiogenic Targeted Therapy Might Be Less Effective in the High-Risk Group

Finally, we investigated the roles of our prognostic model and related lncRNAs in immunotherapy and anti-angiogenic targeted therapy. We first analyzed the expression of four pivotal immune checkpoints (PDCD1, CD274, PDCD1LG2, and CTLA4) in the high- and low-risk groups. There was no significant difference between the two groups (**Figure 6C**). On the other hand, we found that most of the six lncRNAs were not significantly correlated with these immune checkpoints except for DEPDC1-AS1. DEPDC1-AS1 showed positive correlation with PD-1 (*R* = 0.162, *P* = 0.00175), PD-L1 (*R* = 0.136, *P* = 0.019), or CTLA4 (*R* = 0.171, *P* < 0.001) (**Figure 6D**). Therefore, we suggested that the high-risk group might not be sensitive to immunotherapy.

As for the prediction of the efficiency of anti-angiogenic drugs, KDR (VEGFR-2) between the two groups showed a significant difference (*P* = 0.0314), but the VEGFR-2 expression in the low-risk group was higher compared with the high-risk group. In contrast, other anti-angiogenic targets showed no significant changes between the two groups (**Figure 6E**). Besides, only DEPDC1-AS1 and RP11-454K7.3 had a limited correlation with several anti-angiogenic targets (**Figure 6F**). Thus, we suggested that targeted therapy focusing on the above anti-angiogenic targets might be less effective for patients in the high-risk group.

## Discussion

Over recent years, increasing numbers of lncRNAs have been discovered and investigated (32). lncRNAs have a vital role in various cellular and physiologic functions and have been strongly associated with the progression of human cancers (11, 12). For example, MALAT1, one of the most common oncogenic lncRNAs in NSCLC, has been reported to modulate miR-124/STAT3 and promote carcinogenesis (33). Moreover, MALAT1 can enhance epithelial–mesenchymal transition (EMT), increasing metastasis *via* the miR-204/SLUG axis in LUAD and promoting brain metastasis (34, 35). In contrast, another lncRNA, LOC285194, acts as a tumor suppressor that targets p53 and is associated with the KRAS/BRAF/SMEK pathway (36). These strongly emphasize the key roles of lncRNA in cancer biology. However, the prognostic values of lncRNAs in lung adenocarcinoma are still not fully understood. Herein, we first reported these six lncRNAs as potential oncogenes and verified their potential for predicting lung adenocarcinoma.

To date, various prognostic gene signatures for lung cancer prognosis have been identified. For example, a six-gene prognostic signature was developed for predicting disease-free survival (DFS) and OS in NSCLC *via* multivariate regression and stratification analyses (37). The AUC values of ROC curves for this six-gene signature predicting DFS were 0.713 in GSE31210, 0.727 in GSE37745, and 0.746 in GSE50081. Another nine-gene signature containing nine glycolysis-related genes was established, and the ROC curve analysis score in this nine-mRNA signature was 0.712 (38). In addition, a 22-gene signature and an 11-gene signature were reported to significantly dichotomize patients with different OS. The two signatures could serve as independent predictors of OS in lung adenocarcinoma and squamous cell carcinoma, respectively, and the AUC values of the risk score were 0.744 for the TCGA-LUAD cohort and 0.684 for the TCGA-LUSC cohorts (39). In our study, we built a LARSO prognostic model for LUAD, which included six novel lncRNAs (DEPDC1-AS1, RP1-290I10.3, RP11-95M5.1, CTD-2124B20.2, CTD-2168K21.1, and RP11-454K7.3). The best AUC values reached 0.77 for the 1-year ROC curve and 0.8 for the 3-year ROC curve in the validation set. Our prognostic model seemed superior to the above gene signatures on predicting OS of LUAD patients from the AUC values.

TNM is the essential prognostic factor for predicting lung cancer survival time and recurrence rates in the clinic, followed by indexes like sex, age, histological grade, and performance status (40). In our study, the LARSO consisting of six lncRNAs was correlated with performance status and TNM stage. A high LARSO value indicated a more severe stage. The AUC values of this model reached 0.78 in the 1-year ROC curve and 0.77 in the 3-year ROC curve in the validation set, suggesting that LARSO had a more effective performance for OS prediction. Therefore, a comprehensive examination of LUAD patients with high LARSO values should be performed to determine whether they have lymph node metastasis and distant metastasis. Moreover, the AUC value of NRS in the 3-year ROC curve could reach 0.8, further indicating that LARSO combined with stages and N stages could better predict the prognosis of LUAD patients compared with LARSO alone, which suggested to us that in the process of applying our prognostic model, if the clinical staging and N staging of patients cannot be clearly defined, such as the radiological examinations of patients could not determine their stage and they could not be further examined because of their poor basic condition or surgical contraindications, the LARSO could be used for the prediction of prognosis of the patients, while these patients who can obtain a clear stage and N stage could be predicted by the NRS. Due to the small sample size (48 pairs) and potential experimental errors involved in qRT-PCR, the prognostic values of the LARSO and NRS model were similar in the validation set. However, we built the NRS prognostic model by combing LARSO with stage score and N score, which showed a desirable capability for predicting the overall survival of LUAD patients, and the AUC values in NRS were improved compared with the LARSO prognostic model.

Our analysis of the GSEA and KEGG pathway found that these six lncRNAs were most correlated with cell cycle, DNA damage repair, or tumor cell metabolism, which provides hints for the molecular mechanism study about these lncRNAs in the future. As the fundamental requirement for homeostasis, the cell cycle has a vital role in tumor progression, mainly through cell-cycle kinases (cdks) (41), whereas DNA damage-response or DNA-repair genes with germline aberrations induce cancerous tendencies (42). Furthermore, the proliferation and metastasis of tumor cells are strongly influenced by surrounding cells in the tumor microenvironment. The communication between tumor cells and the surrounding cells, such as immune cells, mainly depends on the tumor and correlated cell metabolism (43).

Over the last decade, immunotherapy emerged and greatly changed the landscape of cancer therapy. Immune checkpoint inhibitors for targeted therapy such as anti-PD-1/PD-L1 have shown to be safe and effective against a certain type of cancer (44). In this study, we examined the role of six lncRNAs in tumor immune regulation. However, no significant correlation was observed between these lncRNAs and immune genes or immune-infiltrated cells. Therefore, we concluded that the high-risk group showing high LARSO values might not be sensitive to immunotherapy. In addition, an analysis of immune checkpoint expression between the high- and low-risk groups was also performed. Consistently, there was no significant change of immune checkpoint expression in the two groups, implying that current clinical immunotherapy with immune checkpoint inhibitors might be inefficient for LUAD patients with high LARSO values. Angiogenesis has a critical role in the progression and invasion of cancer cells. Anti-angiogenesis therapy, particularly anti-VEGF therapy, has shown to be effective against several tumors (45). In this study, we found a higher VEGFR-2 expression in the low-risk group than in the high-risk group (**Figure 6E**, *P* = 0.0314). Meanwhile, other anti-angiogenesis targets such as FGFR1, 2, 3, and 4 showed no significant change between the two groups. Thus, we concluded that LUAD patients with high LARSO values might not benefit from anti-angiogenesis targets and that alternative therapies are required.

This study has a few limitations. Firstly, no specific LARSO cutoff value was determined to define the high- and low-risk groups due to calculation variance of RNA-seq data or qRT-PCR. To solve this problem, LARSO cutoff values should be obtained in tests of a small set of samples and verified by large prospective clinical studies. Secondly, although we preliminarily verified the prognostic value of the LARSO model by using qRT-PCR assay, the sample size (48 pairs) was small. So, the value of this model needs to be further verified in large-scale clinical trials. Thirdly, the molecular mechanism, including cell cycle regulation, DNA damage repair, or tumor cell metabolism regulation, of the current model and six lncRNAs was not further investigated in the current study. In the future, we plan to investigate the roles of these lncRNAs based on *in-vivo* and *in-vitro* experiments referring to GSEA results. Finally, the LUAD patients we collected for validation were all diagnosed at stage IA–IIIB and feasible for surgery, so the efficiency of immunotherapy drugs and anti-angiogenic drugs were lacking for further evaluation. Nevertheless, analysis results of immune checkpoints or anti-angiogenesis targets in the LARSO high- and low-risk groups can still provide theoretical support for predicting the efficiency of correlated drugs. Also, further clinical trials need to be performed to verify whether high LARSO can be used as an indicator of drug resistance in immunotherapy as well as anti-angiogenesis therapy.

## Conclusion

Six lncRNAs were identified by integrated bioinformatics analysis and further validated using clinical samples. These six lncRNAs have shown to be potential oncogenic and predictive factors of LUAD; a positive correlation was found between the risk of death and lncRNA expression. Furthermore, a prognostic signature was defined based on these six lncRNAs, showing adequate reliability and sensitivity in our study. In addition, we demonstrated that LARSO combined with stages and N stages could better predict the prognosis of LUAD patients compared with LARSO alone. These findings provide the theoretical basis for effective promotion and exploration of potential biomarkers for predicting LUAD prognosis.

## Data Availability Statement

Publicly available datasets were analyzed in this study. These data can be found here: The Cancer Genome Atlas (TCGA) database (https://portal.gdc.cancer.gov).

## Ethics Statement

The studies involving human participants were reviewed and approved by the Medical Ethics Committee of the Second Affiliated Hospital of Zhejiang University School of Medicine. The patients/participants provided their written informed consent to participate in this study.

## Author Contributions

Conceptualization: KW and WC. Data curation: LY and YW. Formal analysis: LY, YW, and HX. Methodology: LY, YW, and LZ. Software: LY, YW, and HX. Supervision: KW and WC. Validation: LY, YW, JZ, and YFW. Visualization: LY, YW, HX, XZ, and YFW. Writing—original draft: LY and YW. Writing—review and editing: KW and WC. All authors contributed to the article and approved the submitted version.

## Funding

This work was supported by the National Natural Science Foundation of China (grants 81902331 and 81871874) and the Zhejiang Provincial Key Research and Development Project (No. 2020C03027).

## Conflict of Interest

The authors declare that the research was conducted in the absence of any commercial or financial relationships that could be construed as a potential conflict of interest.

## Publisher’s Note

All claims expressed in this article are solely those of the authors and do not necessarily represent those of their affiliated organizations, or those of the publisher, the editors and the reviewers. Any product that may be evaluated in this article, or claim that may be made by its manufacturer, is not guaranteed or endorsed by the publisher.
