# Supplementary material for: Identification and Validation of a Novel Six-lncRNA-Based Prognostic Model for Lung Adenocarcinoma
Source: Front Oncol. 2022 Jan 17;11:775583. doi: 10.3389/fonc.2021.775583 (PMC8801419; doi:10.3389/fonc.2021.775583)
Supplement: Supplementary file 1 [file DataSheet_1.pdf]

# *Supplementary Material*

## Supplementary Tables

**Table S1.** Primer sequences for q-PCR

| Primer name | Primer sequence (5 '→ 3') |
|---|---|
| h-β-actin-F | GGGAAATCGTGCGTGACATTAAG |
| h-β-actin-R | TGTGTTGGCGTACAGGTCTTTG |
| h-CTD-2124B20.2-F1 | TTCCCTATTTGGCAGGATAGTC |
| h-CTD-2124B20.2-R1 | AAGTTCCAGGCAGCTCGCT |
| h-CTD-2168K21.1-F1 | TGCTGGATTTAGAGGTGGTGTATT |
| h-CTD-2168K21.1-R1 | CCCTGAGAGTGTTCTCTTTCCTT |
| h-DEPDC1-AS1-F1 | GGGAGCGGATAAGGAAGTCA |
| h-DEPDC1-AS1-R1 | TTAGAGGAGTGCCAGGATTGTAG |
| h-RP1-290I10.3-_F1 | CTGCTGTCAGTTGAAAGCGTG |
| h-RP1-290I10.3-_R1 | GAAGGTGAAGAAGGAAGGGTGT |
| h-RP11-454K7.3-F1 | ATGTGGATAGTTCTGAATGTGCC |
| h-RP11-454K7.3-R1 | CAAACCACAGACATAGCCAACC |
| h-RP11-95M5.1-F1 | CACACAGGTAACTGTCGGCAA |
| h-RP11-95M5.1-R1 | AATGGCACGTGGATTAGATCTC |

**Table S2.** The GSEA results of hallmark and KEGG pathway enrichment analysis base on the cutoff of LARSO=0.110 in the modeling set

| Term | ES | NES | NP | FDR | FWER |
|---|---|---|---|---|---|
| HALLMARK_G2M_CHECKPOINT | -0.8037 | -2.0907 | 0 | 0.0084 | 0.006 |
| HALLMARK_MTORC1_SIGNALING | -0.7035 | -2.0793 | 0 | 0.0051 | 0.007 |
| HALLMARK_E2F_TARGETS | -0.8342 | -2.0111 | 0 | 0.0119 | 0.021 |
| HALLMARK_MYC_TARGETS_V1 | -0.794 | -1.9831 | 0.002 | 0.0123 | 0.029 |
| HALLMARK_DNA_REPAIR | -0.6495 | -1.8789 | 0.004 | 0.0322 | 0.074 |
| HALLMARK_GLYCOLYSIS | -0.5627 | -1.8562 | 0.0086 | 0.0338 | 0.087 |
| HALLMARK_OXIDATIVE_PHOSPHORYLATION | -0.667 | -1.8004 | 0.0176 | 0.0467 | 0.125 |
| HALLMARK_MYC_TARGETS_V2 | -0.7832 | -1.7896 | 0.0084 | 0.0445 | 0.13 |
| HALLMARK_REACTIVE_OXYGEN_SPECIES_PATHWAY | -0.6331 | -1.7753 | 0.0104 | 0.0445 | 0.144 |
| HALLMARK_UNFOLDED_PROTEIN_RESPONSE | -0.5758 | -1.6975 | 0.0442 | 0.0696 | 0.21 |
| HALLMARK_PI3K_AKT_MTOR_SIGNALING | -0.5179 | -1.6932 | 0.0339 | 0.0647 | 0.213 |
| HALLMARK_SPERMATOGENESIS | -0.4329 | -1.6446 | 0.0254 | 0.0815 | 0.266 |
| HALLMARK_MITOTIC_SPINDLE | -0.5529 | -1.6277 | 0.0705 | 0.0827 | 0.282 |
| HALLMARK_FATTY_ACID_METABOLISM | -0.4795 | -1.6203 | 0.0328 | 0.08 | 0.289 |
| HALLMARK_PROTEIN_SECRETION | -0.5531 | -1.5902 | 0.084 | 0.0889 | 0.322 |
| HALLMARK_INTERFERON_ALPHA_RESPONSE | -0.6482 | -1.5796 | 0.0992 | 0.089 | 0.335 |
| HALLMARK_UV_RESPONSE_UP | -0.4487 | -1.576 | 0.032 | 0.0858 | 0.338 |

| Term | ES | NES | NP | FDR | FWER |
|---|---|---|---|---|---|
| HALLMARK_CHOLESTEROL_HOMEOSTASIS | -0.493 | -1.5641 | 0.0473 | 0.0868 | 0.356 |
| HALLMARK_ADIPOGENESIS | -0.4616 | -1.4931 | 0.0698 | 0.1194 | 0.428 |
| HALLMARK_ESTROGEN_RESPONSE_LATE | -0.3858 | -1.4703 | 0.0471 | 0.1253 | 0.444 |
| HALLMARK_ANDROGEN_RESPONSE | -0.4304 | -1.4386 | 0.0998 | 0.1379 | 0.469 |
| HALLMARK_PEROXISOME | -0.3947 | -1.3526 | 0.1157 | 0.1882 | 0.551 |
| HALLMARK_HYPOXIA | -0.3906 | -1.3412 | 0.1406 | 0.1874 | 0.562 |
| HALLMARK_XENOBIOTIC_METABOLISM | -0.3444 | -1.3205 | 0.11 | 0.1957 | 0.586 |
| HALLMARK_INTERFERON_GAMMA_RESPONSE | -0.4738 | -1.2756 | 0.259 | 0.2202 | 0.628 |
| HALLMARK_APOPTOSIS | -0.3697 | -1.2186 | 0.2086 | 0.2576 | 0.679 |
| HALLMARK_APICAL_JUNCTION | -0.3324 | -1.1192 | 0.3389 | 0.3395 | 0.746 |
| HALLMARK_ESTROGEN_RESPONSE_EARLY | -0.2957 | -1.0738 | 0.3482 | 0.3755 | 0.773 |
| HALLMARK_HEME_METABOLISM | -0.2737 | -1.0035 | 0.4338 | 0.4432 | 0.825 |
| HALLMARK_P53_PATHWAY | -0.2893 | -0.9877 | 0.464 | 0.4469 | 0.836 |
| HALLMARK_APICAL_SURFACE | -0.2964 | -0.9808 | 0.4617 | 0.4408 | 0.839 |
| HALLMARK_COMPLEMENT | -0.2747 | -0.9108 | 0.5261 | 0.5185 | 0.873 |
| HALLMARK_WNT_BETA_CATENIN_SIGNALING | -0.2791 | -0.8641 | 0.6092 | 0.5617 | 0.885 |
| HALLMARK_PANCREAS_BETA_CELLS | -0.2365 | -0.8021 | 0.721 | 0.6411 | 0.909 |
| HALLMARK_TGF_BETA_SIGNALING | -0.2762 | -0.7973 | 0.6582 | 0.6305 | 0.911 |
| HALLMARK_ANGIOGENESIS | -0.2587 | -0.7735 | 0.715 | 0.6523 | 0.919 |

| Term | ES | NES | NP | FDR | FWER |
|---|---|---|---|---|---|
| HALLMARK_UV_RESPONSE_DN | -0.2263 | -0.7523 | 0.7339 | 0.6657 | 0.923 |
| HALLMARK_EPITHELIAL_MESENCHYMAL_TRANSITION | -0.2652 | -0.7293 | 0.6609 | 0.6794 | 0.928 |
| HALLMARK_NOTCH_SIGNALING | 0.2231 | 0.6484 | 0.8874 | 0.8092 | 0.977 |
| HALLMARK_HEDGEHOG_SIGNALING | 0.2272 | 0.6527 | 0.8705 | 0.8764 | 0.977 |
| HALLMARK_MYOGENESIS | 0.2146 | 0.7194 | 0.8163 | 0.8527 | 0.968 |
| HALLMARK_COAGULATION | 0.2376 | 0.7481 | 0.7648 | 0.8901 | 0.962 |
| HALLMARK_TNFA_SIGNALING_VIA_NFKB | 0.2536 | 0.752 | 0.7287 | 0.9914 | 0.961 |
| HALLMARK_ALLOGRAFT_REJECTION | 0.2787 | 0.816 | 0.6367 | 0.9793 | 0.944 |
| HALLMARK_IL2_STAT5_SIGNALING | 0.2431 | 0.8259 | 0.6984 | 1 | 0.937 |
| HALLMARK_KRAS_SIGNALING_DN | 0.2241 | 0.8284 | 0.705 | 1 | 0.935 |
| HALLMARK_INFLAMMATORY_RESPONSE | 0.2918 | 0.9108 | 0.5526 | 1 | 0.897 |
| HALLMARK_BILE_ACID_METABOLISM | 0.2662 | 0.9109 | 0.5731 | 1 | 0.897 |
| HALLMARK_KRAS_SIGNALING_UP | 0.264 | 0.9275 | 0.5109 | 1 | 0.893 |
| HALLMARK_IL6_JAK_STAT3_SIGNALING | 0.359 | 1.0348 | 0.4175 | 1 | 0.838 |
| KEGG_PROTEASOME | -0.8816 | -2.0192 | 0 | 0.0495 | 0.032 |
| KEGG_CELL_CYCLE | -0.7312 | -2.0036 | 0 | 0.034 | 0.042 |
| KEGG_NUCLEOTIDE_EXCISION_REPAIR | -0.7589 | -1.9992 | 0 | 0.0237 | 0.044 |
| KEGG_MISMATCH_REPAIR | -0.8499 | -1.918 | 0 | 0.058 | 0.11 |
| KEGG_OOCYTE_MEIOSIS | -0.5855 | -1.8699 | 0.0022 | 0.0843 | 0.158 |

| Term | ES | NES | NP | FDR | FWER |
|---|---|---|---|---|---|
| KEGG_PENTOSE_PHOSPHATE_PATHWAY | -0.671 | -1.862 | 0.0088 | 0.0772 | 0.166 |
| KEGG_DNA_REPLICATION | -0.8587 | -1.8599 | 0 | 0.0674 | 0.167 |
| KEGG_CITRATE_CYCLE_TCA_CYCLE | -0.7459 | -1.8126 | 0.006 | 0.0949 | 0.237 |
| KEGG_PYRIMIDINE_METABOLISM | -0.6105 | -1.8038 | 0.0106 | 0.0929 | 0.256 |
| KEGG_HOMOLOGOUS_RECOMBINATION | -0.7536 | -1.7967 | 0.0084 | 0.0898 | 0.265 |
| KEGG_GLYCOLYSIS_GLUCONEOGENESIS | -0.541 | -1.7527 | 0.0119 | 0.1247 | 0.335 |
| KEGG_PORPHYRIN_AND_CHLOROPHYLL_METABOLISM | -0.5316 | -1.7424 | 0.0176 | 0.1229 | 0.354 |
| KEGG_SPLICEOSOME | -0.6724 | -1.7315 | 0.0351 | 0.1231 | 0.373 |
| KEGG_CYSTEINE_AND_METHIONINE_METABOLISM | -0.5532 | -1.7288 | 0.0046 | 0.1166 | 0.374 |
| KEGG_PATHOGENIC_ESCHERICHIA_COLI_INFECTION | -0.5809 | -1.7272 | 0.0132 | 0.1101 | 0.378 |
| KEGG_AMINOACYL_TRNA_BIOSYNTHESIS | -0.7003 | -1.7015 | 0.0277 | 0.1292 | 0.434 |
| KEGG_P53_SIGNALING_PATHWAY | -0.5278 | -1.6977 | 0.0087 | 0.1239 | 0.438 |
| KEGG_RNA_DEGRADATION | -0.5838 | -1.6783 | 0.0356 | 0.1368 | 0.482 |
| KEGG_BASAL_TRANSCRIPTION_FACTORS | -0.6198 | -1.677 | 0.0333 | 0.1308 | 0.483 |
| KEGG_TERPENOID_BACKBONE_BIOSYNTHESIS | -0.7324 | -1.6693 | 0.0426 | 0.1314 | 0.499 |
| KEGG_PARKINSONS_DISEASE | -0.5933 | -1.6593 | 0.0368 | 0.1353 | 0.517 |
| KEGG_N_GLYCAN_BIOSYNTHESIS | -0.5879 | -1.6524 | 0.0393 | 0.1345 | 0.527 |
| KEGG_PROGESTERONE_MEDIATED_OOCYTE_MATURATION | -0.5144 | -1.646 | 0.0343 | 0.1347 | 0.539 |

| Term | ES | NES | NP | FDR | FWER |
|---|---|---|---|---|---|
| KEGG_PROTEIN_EXPORT | -0.6821 | -1.6359 | 0.0395 | 0.1375 | 0.556 |
| KEGG_PYRUVATE_METABOLISM | -0.5401 | -1.6153 | 0.0591 | 0.1485 | 0.582 |
| KEGG_PHENYLALANINE_METABOLISM | -0.5711 | -1.605 | 0.0405 | 0.1532 | 0.6 |
| KEGG_ONE_CARBON_POOL_BY_FOLATE | -0.643 | -1.6046 | 0.0406 | 0.1483 | 0.601 |
| KEGG_HUNTINGTONS_DISEASE | -0.5073 | -1.5902 | 0.0357 | 0.1555 | 0.622 |
| KEGG_RNA_POLYMERASE | -0.6108 | -1.5742 | 0.0696 | 0.166 | 0.645 |
| KEGG_GLYOXYLATE_AND_DICARBOXYLATE_METABOLISM | -0.6313 | -1.5725 | 0.0413 | 0.1624 | 0.647 |
| KEGG_UBIQUITIN_MEDIATED_PROTEOLYSIS | -0.5081 | -1.5699 | 0.0719 | 0.1595 | 0.656 |
| KEGG_BASE_EXCISION_REPAIR | -0.641 | -1.5683 | 0.0619 | 0.1558 | 0.657 |
| KEGG_BIOSYNTHESIS_OF_UNSATURATED_FATTY_ACIDS | -0.6018 | -1.5664 | 0.0545 | 0.1525 | 0.659 |
| KEGG_OXIDATIVE_PHOSPHORYLATION | -0.5705 | -1.5595 | 0.0639 | 0.1537 | 0.664 |
| KEGG_LYSINE_DEGRADATION | -0.5509 | -1.5493 | 0.0594 | 0.1587 | 0.678 |
| KEGG_PURINE_METABOLISM | -0.4439 | -1.5352 | 0.0424 | 0.166 | 0.701 |
| KEGG_ALZHEIMERS_DISEASE | -0.4747 | -1.5308 | 0.0496 | 0.1652 | 0.706 |
| KEGG_FOLATE_BIOSYNTHESIS | -0.6211 | -1.489 | 0.0775 | 0.1996 | 0.752 |
| KEGG_VALINE_LEUCINE_AND_ISOLEUCINE_BIOSYNTHESIS | -0.6845 | -1.4852 | 0.1022 | 0.1986 | 0.756 |
| KEGG_GLUTATHIONE_METABOLISM | -0.4995 | -1.4782 | 0.0843 | 0.2009 | 0.765 |
| KEGG_BUTANOATE_METABOLISM | -0.5103 | -1.477 | 0.1 | 0.1972 | 0.767 |
| KEGG_PANCREATIC_CANCER | -0.4772 | -1.473 | 0.1019 | 0.1966 | 0.775 |

| Term | ES | NES | NP | FDR | FWER |
|---|---|---|---|---|---|
| KEGG_NON_HOMOLOGOUS_END_JOINING | -0.6412 | -1.4433 | 0.0996 | 0.2202 | 0.799 |
| KEGG_GALACTOSE_METABOLISM | -0.5001 | -1.4422 | 0.0926 | 0.2164 | 0.8 |
| KEGG_SMALL_CELL_LUNG_CANCER | -0.4556 | -1.4369 | 0.1041 | 0.2163 | 0.802 |
| KEGG_PROPANOATE_METABOLISM | -0.5275 | -1.4367 | 0.1164 | 0.2118 | 0.802 |
| KEGG_DRUG_METABOLISM_OTHER_ENZYMES | -0.4002 | -1.4324 | 0.0571 | 0.212 | 0.809 |
| KEGG_THYROID_CANCER | -0.4908 | -1.4142 | 0.1265 | 0.2246 | 0.819 |
| KEGG_AMINO_SUGAR_AND_NUCLEOTIDE_SUGAR_METABOLISM | -0.4778 | -1.383 | 0.1349 | 0.2515 | 0.844 |
| KEGG_PROSTATE_CANCER | -0.4234 | -1.3797 | 0.1224 | 0.2504 | 0.849 |
| KEGG_RENAL_CELL_CARCINOMA | -0.4527 | -1.3751 | 0.1529 | 0.2503 | 0.852 |
| KEGG_ALANINE_ASPARTATE_AND_GLUTAMATE_METABOLISM | -0.4228 | -1.3496 | 0.1308 | 0.2731 | 0.876 |
| KEGG_ADHERENS_JUNCTION | -0.4502 | -1.3247 | 0.1918 | 0.2948 | 0.889 |
| KEGG_BLADDER_CANCER | -0.4172 | -1.2906 | 0.1689 | 0.33 | 0.912 |
| KEGG_PEROXISOME | -0.4187 | -1.2897 | 0.214 | 0.3249 | 0.912 |
| KEGG_SULFUR_METABOLISM | -0.509 | -1.2813 | 0.1849 | 0.329 | 0.912 |
| KEGG_GLYCINE_SERINE_AND_THREONINE_METABOLISM | -0.4115 | -1.2753 | 0.1937 | 0.3306 | 0.915 |
| KEGG_AMYOTROPHIC_LATERAL_SCLEROSIS_ALS | -0.3745 | -1.2472 | 0.182 | 0.3607 | 0.926 |
| KEGG_PATHWAYS_IN_CANCER | -0.3388 | -1.2463 | 0.2119 | 0.3558 | 0.926 |
| KEGG_BETA_ALANINE_METABOLISM | -0.4112 | -1.2451 | 0.2185 | 0.3511 | 0.926 |
| KEGG_TRYPTOPHAN_METABOLISM | -0.3742 | -1.223 | 0.206 | 0.374 | 0.932 |

| Term | ES | NES | NP | FDR | FWER |
| --- | --- | --- | --- | --- | --- |
| KEGG_NICOTINATE_AND_NICOTINAMIDE_METABOLISM | -0.3994 | -1.22 | 0.2206 | 0.3722 | 0.934 |
| KEGG_CHRONIC_MYELOID_LEUKEMIA | -0.4084 | -1.2183 | 0.2429 | 0.3682 | 0.934 |
| KEGG_CARDIAC_MUSCLE_CONTRACTION | -0.3383 | -1.2171 | 0.2268 | 0.3643 | 0.935 |
| KEGG_GLIOMA | -0.377 | -1.2168 | 0.2419 | 0.3591 | 0.935 |
| KEGG_GLYCOSYLPHOSPHATIDYLINOSITOL_GPI_ANCHOR_BIOSYNTHESIS | -0.459 | -1.2105 | 0.2673 | 0.3614 | 0.939 |
| KEGG_ANTIGEN_PROCESSING_AND_PRESENTATION | -0.4114 | -1.2031 | 0.291 | 0.3651 | 0.943 |
| KEGG_PANTOTHENATE_AND_COA_BIOSYNTHESIS | -0.4162 | -1.1948 | 0.215 | 0.3712 | 0.951 |
| KEGG_STARCH_AND_SUCROSE_METABOLISM | -0.3305 | -1.1903 | 0.1994 | 0.3713 | 0.951 |
| KEGG_VALINE_LEUCINE_AND_ISOLEUCINE_DEGRADATION | -0.4393 | -1.1887 | 0.2714 | 0.3679 | 0.953 |
| KEGG_REGULATION_OF_ACTIN_CYTOSKELETON | -0.3359 | -1.1861 | 0.2484 | 0.366 | 0.954 |
| KEGG_MELANOMA | -0.3359 | -1.1858 | 0.2354 | 0.3613 | 0.954 |
| KEGG_LIMONENE_AND_PINENE_DEGRADATION | -0.5616 | -1.1837 | 0.31 | 0.3588 | 0.955 |
| KEGG_TYROSINE_METABOLISM | -0.3531 | -1.1825 | 0.2197 | 0.3555 | 0.955 |
| KEGG_ERBB_SIGNALING_PATHWAY | -0.3551 | -1.1627 | 0.2758 | 0.375 | 0.965 |
| KEGG_VIBRIO_CHOLERAE_INFECTION | -0.3715 | -1.1598 | 0.2887 | 0.3739 | 0.966 |
| KEGG_GAP_JUNCTION | -0.3418 | -1.1585 | 0.2719 | 0.3708 | 0.966 |
| KEGG_TIGHT_JUNCTION | -0.3243 | -1.1552 | 0.2966 | 0.3704 | 0.966 |

| Term | ES | NES | NP | FDR | FWER |
|---|---|---|---|---|---|
| KEGG_GLYCOSPHINGOLIPID_BIOSYNTHESIS_LACTO_AND_NEOLACTO_SERIES | -0.3858 | -1.1455 | 0.2697 | 0.3783 | 0.969 |
| KEGG_NOTCH_SIGNALING_PATHWAY | -0.3947 | -1.1348 | 0.3252 | 0.3872 | 0.973 |
| KEGG_PENTOSE_AND_GLUCURONATE_INTERCONVERSIONS | -0.3628 | -1.1321 | 0.3145 | 0.3855 | 0.973 |
| KEGG_ENDOCYTOSIS | -0.3447 | -1.125 | 0.3096 | 0.3898 | 0.973 |
| KEGG_ASCORBATE_AND_ALDARATE_METABOLISM | -0.3751 | -1.122 | 0.2952 | 0.3889 | 0.973 |
| KEGG_EPITHELIAL_CELL_SIGNALING_IN_HELICOBACTER_PYLORI_INFECTION | -0.3536 | -1.1168 | 0.3028 | 0.3913 | 0.974 |
| KEGG_STEROID_BIOSYNTHESIS | -0.4568 | -1.1158 | 0.3471 | 0.3877 | 0.975 |
| KEGG_PRION_DISEASES | -0.36 | -1.0713 | 0.3621 | 0.4383 | 0.985 |
| KEGG_WNT_SIGNALING_PATHWAY | -0.3007 | -1.0613 | 0.3521 | 0.4472 | 0.988 |
| KEGG_FRUCTOSE_AND_MANNOSE_METABOLISM | -0.3592 | -1.0503 | 0.3996 | 0.4574 | 0.99 |
| KEGG_ARGININE_AND_PROLINE_METABOLISM | -0.3265 | -1.0426 | 0.385 | 0.4625 | 0.99 |
| KEGG_FOCAL_ADHESION | -0.3175 | -1.0343 | 0.4129 | 0.4699 | 0.992 |
| KEGG_ENDOMETRIAL_CANCER | -0.3316 | -0.9857 | 0.4575 | 0.5335 | 0.996 |
| KEGG_COLORECTAL_CANCER | -0.3179 | -0.9847 | 0.4592 | 0.529 | 0.996 |
| KEGG_FC_GAMMA_R_MEDIATED_PHAGOCYTOSIS | -0.3077 | -0.9816 | 0.472 | 0.5285 | 0.996 |
| KEGG_APOPTOSIS | -0.3009 | -0.9774 | 0.4909 | 0.529 | 0.996 |
| KEGG_RIG_I_LIKE_RECEPTOR_SIGNALING_PATHWAY | -0.296 | -0.9773 | 0.4706 | 0.5239 | 0.996 |
| KEGG_AXON_GUIDANCE | -0.2793 | -0.9759 | 0.4521 | 0.5205 | 0.996 |

| Term | ES | NES | NP | FDR | FWER |
|---|---|---|---|---|---|
| KEGG_NON_SMALL_CELL_LUNG_CANCER | -0.3144 | -0.9734 | 0.4939 | 0.5189 | 0.996 |
| KEGG_FATTY_ACID_METABOLISM | -0.3332 | -0.966 | 0.4511 | 0.5245 | 0.998 |
| KEGG_ADIPOCYTOKINE_SIGNALING_PATHWAY | -0.2761 | -0.9407 | 0.5486 | 0.5553 | 0.998 |
| KEGG_RIBOFLAVIN_METABOLISM | -0.3401 | -0.924 | 0.5396 | 0.5752 | 0.998 |
| KEGG_DORSO_VENTRAL_AXIS_FORMATION | -0.3289 | -0.9171 | 0.5586 | 0.5802 | 0.998 |
| KEGG_NEUROTROPHIN_SIGNALING_PATHWAY | -0.2736 | -0.8997 | 0.5549 | 0.6013 | 0.998 |
| KEGG_RETINOL_METABOLISM | -0.2505 | -0.8935 | 0.5884 | 0.6053 | 0.998 |
| KEGG_CYTOSOLIC_DNA_SENSING_PATHWAY | -0.2841 | -0.8917 | 0.5832 | 0.6023 | 0.998 |
| KEGG_LONG_TERM_POTENTIATION | -0.2456 | -0.8542 | 0.6198 | 0.6537 | 0.998 |
| KEGG_SNARE_INTERACTIONS_IN_VESICULAR_TRANSPORT | -0.2907 | -0.8488 | 0.6392 | 0.6554 | 0.998 |
| KEGG_SELENOAMINO_ACID_METABOLISM | -0.3093 | -0.8437 | 0.6454 | 0.6568 | 0.998 |
| KEGG_STEROID_HORMONE_BIOSYNTHESIS | -0.232 | -0.8381 | 0.6655 | 0.6604 | 0.999 |
| KEGG_VASOPRESSIN_REGULATED_WATER_REABSORPTION | -0.2824 | -0.8379 | 0.6329 | 0.6546 | 0.999 |
| KEGG_PPAR_SIGNALING_PATHWAY | -0.2343 | -0.8365 | 0.683 | 0.6508 | 0.999 |
| KEGG_VIRAL_MYOCARDITIS | -0.2847 | -0.8243 | 0.6394 | 0.6643 | 0.999 |
| KEGG_TOLL_LIKE_RECEPTOR_SIGNALING_PATHWAY | -0.2473 | -0.7871 | 0.7052 | 0.7162 | 1 |
| KEGG_SPHINGOLIPID_METABOLISM | -0.2629 | -0.7825 | 0.7167 | 0.7173 | 1 |

| Term | ES | NES | NP | FDR | FWER |
|---|---|---|---|---|---|
| KEGG_ACUTE_MYELOID_LEUKEMIA | -0.2617 | -0.7823 | 0.7129 | 0.7112 | 1 |
| KEGG_SYSTEMIC_LUPUS_ERYTHEMATOSUS | -0.2478 | -0.7804 | 0.6414 | 0.7078 | 1 |
| KEGG_GLYCOSAMINOGLYCAN_BIOSYNTHESIS_KERATAN_SULFATE | -0.3073 | -0.7776 | 0.7483 | 0.7061 | 1 |
| KEGG_NATURAL_KILLER_CELL_MEDIATED_CYTOTOXICITY | -0.2269 | -0.7454 | 0.7457 | 0.748 | 1 |
| KEGG_INSULIN_SIGNALING_PATHWAY | -0.2103 | -0.7432 | 0.7935 | 0.7448 | 1 |
| KEGG_MELANOGENESIS | -0.2017 | -0.7317 | 0.8534 | 0.7563 | 1 |
| KEGG_GLYCEROLIPID_METABOLISM | -0.1855 | -0.6599 | 0.9608 | 0.8501 | 1 |
| KEGG_GLYCOSAMINOGLYCAN_BIOSYNTHESIS_CHONDROITIN_SULFATE | -0.235 | -0.619 | 0.8983 | 0.8901 | 1 |
| KEGG_CIRCADIAN_RHYTHM_MAMMAL | -0.2444 | -0.5837 | 0.9173 | 0.9178 | 1 |
| KEGG_MATURITY_ONSET_DIABETES_OF_THE_YOUNG | -0.1959 | -0.578 | 0.9636 | 0.9151 | 1 |
| KEGG_RIBOSOME | 0.1855 | 0.3814 | 0.9283 | 0.9975 | 1 |
| KEGG_GLYCOSAMINOGLYCAN_DEGRADATION | 0.1946 | 0.5138 | 0.9745 | 0.9814 | 1 |
| KEGG_OLFACTORY_TRANSDUCTION | 0.1118 | 0.5414 | 0.9983 | 0.9815 | 1 |
| KEGG_GLYCOSAMINOGLYCAN_BIOSYNTHESIS_HEPARAN_SULFATE | 0.2042 | 0.5653 | 0.9497 | 0.9798 | 1 |
| KEGG_TGF_BETA_SIGNALING_PATHWAY | 0.2098 | 0.682 | 0.8589 | 0.8595 | 1 |
| KEGG_METABOLISM_OF_XENOBIOTICS_BY_CYTOCHROME_P450 | 0.2505 | 0.7226 | 0.7789 | 0.8143 | 1 |
| KEGG_REGULATION_OF_AUTOPHA | 0.2212 | 0.7481 | 0.8172 | 0.7886 | 1 |

| Term | ES | NES | NP | FDR | FWER |
| --- | --- | --- | --- | --- | --- |
| GY | | | | | |
| KEGG_GLYCOSPHINGOLIPID_BIOSYNTHESIS_GLOBO_SERIES | 0.3078 | 0.7483 | 0.7942 | 0.8023 | 1 |
| KEGG_CALCIUM_SIGNALING_PATHWAY | 0.2072 | 0.7498 | 0.8168 | 0.8143 | 1 |
| KEGG_MTOR_SIGNALING_PATHWAY | 0.2387 | 0.7504 | 0.7778 | 0.8285 | 1 |
| KEGG_GLYCOSPHINGOLIPID_BIOSYNTHESIS_GANGLIO_SERIES | 0.2998 | 0.7537 | 0.76 | 0.8388 | 1 |
| KEGG_O_GLYCAN_BIOSYNTHESIS | 0.2758 | 0.7549 | 0.7491 | 0.8527 | 1 |
| KEGG_HISTIDINE_METABOLISM | 0.2646 | 0.7565 | 0.7906 | 0.8665 | 1 |
| KEGG_LYSOSOME | 0.257 | 0.7634 | 0.7093 | 0.8715 | 1 |
| KEGG_MAPK_SIGNALING_PATHWAY | 0.2147 | 0.7643 | 0.7524 | 0.8876 | 1 |
| KEGG_BASAL_CELL_CARCINOMA | 0.2489 | 0.7722 | 0.728 | 0.8909 | 1 |
| KEGG_ECM_RECEPTOR_INTERACTION | 0.2782 | 0.7778 | 0.6949 | 0.8995 | 1 |
| KEGG_HEDGEHOG_SIGNALING_PATHWAY | 0.252 | 0.7911 | 0.695 | 0.8939 | 1 |
| KEGG_PRIMARY_IMMUNODEFICIENCY | 0.322 | 0.7943 | 0.6528 | 0.9067 | 1 |
| KEGG_INOSITOL_PHOSPHATE_METABOLISM | 0.279 | 0.8581 | 0.6171 | 0.8031 | 0.999 |
| KEGG_ARRHYTHMOGENIC_RIGHT_VENTRICULAR_CARDIOMYOPATHY_ARVC | 0.2727 | 0.8625 | 0.6219 | 0.8128 | 0.999 |
| KEGG_OTHER_GLYCAN_DEGRADATION | 0.3694 | 0.8887 | 0.5781 | 0.7827 | 0.999 |
| KEGG_VEGF_SIGNALING_PATHWAY | 0.2625 | 0.8901 | 0.5943 | 0.7988 | 0.999 |

| Term | ES | NES | NP | FDR | FWER |
|---|---|---|---|---|---|
| KEGG_LONG_TERM_DEPRESSION | 0.2623 | 0.9001 | 0.589 | 0.7998 | 0.999 |
| KEGG_B_CELL_RECEPTOR_SIGNALING_PATHWAY | 0.2937 | 0.9059 | 0.5424 | 0.8089 | 0.999 |
| KEGG_PRIMARY_BILE_ACID_BIOSYNTHESIS | 0.3354 | 0.9081 | 0.5742 | 0.8257 | 0.999 |
| KEGG_DILATED_CARDIOMYOPATHY | 0.2765 | 0.9091 | 0.5699 | 0.8463 | 0.999 |
| KEGG_T_CELL_RECEPTOR_SIGNALING_PATHWAY | 0.2856 | 0.9111 | 0.5347 | 0.8653 | 0.999 |
| KEGG_GNRH_SIGNALING_PATHWAY | 0.2784 | 0.9407 | 0.542 | 0.8258 | 0.998 |
| KEGG_HYPERTROPHIC_CARDIOMYOPATHY_HCM | 0.2858 | 0.9526 | 0.508 | 0.8247 | 0.998 |
| KEGG_DRUG_METABOLISM_CYTOCHROME_P450 | 0.3235 | 0.963 | 0.5022 | 0.8258 | 0.998 |
| KEGG_NOD_LIKE_RECEPTOR_SIGNALING_PATHWAY | 0.3223 | 0.9693 | 0.4868 | 0.8366 | 0.998 |
| KEGG_COMPLEMENT_AND_COAGULATION_CASCADES | 0.3315 | 0.9964 | 0.4607 | 0.8027 | 0.998 |
| KEGG_LEUKOCYTE_TRANSENDOTHELIAL_MIGRATION | 0.3054 | 1.0073 | 0.4291 | 0.8054 | 0.998 |
| KEGG_TYPE_II_DIABETES_MELLITUS | 0.3234 | 1.0277 | 0.4092 | 0.7842 | 0.996 |
| KEGG_TYPE_I_DIABETES_MELLITUS | 0.4043 | 1.0344 | 0.4353 | 0.7964 | 0.995 |
| KEGG_ABC_TRANSPORTERS | 0.337 | 1.0514 | 0.3922 | 0.785 | 0.995 |
| KEGG_GLYCEROPHOSPHOLIPID_METABOLISM | 0.3181 | 1.0641 | 0.3617 | 0.7844 | 0.992 |
| KEGG_PHOSPHATIDYLINOSITOL_SIGNALING_SYSTEM | 0.3349 | 1.0728 | 0.3839 | 0.7951 | 0.992 |
| KEGG_VASCULAR_SMOOTH_MUSCLE_CONTRACTION | 0.3182 | 1.075 | 0.3693 | 0.8229 | 0.992 |

| Term | ES | NES | NP | FDR | FWER |
|---|---|---|---|---|---|
| KEGG_NITROGEN_METABOLISM | 0.3739 | 1.0875 | 0.348 | 0.8268 | 0.991 |
| KEGG_ETHER_LIPID_METABOLISM | 0.3734 | 1.1112 | 0.3192 | 0.8041 | 0.989 |
| KEGG_CHEMOKINE_SIGNALING_PATHWAY | 0.3429 | 1.1165 | 0.3181 | 0.8283 | 0.989 |
| KEGG_FC_EPSILON_RI_SIGNALING_PATHWAY | 0.3426 | 1.1252 | 0.3112 | 0.8459 | 0.989 |
| KEGG_JAK_STAT_SIGNALING_PATHWAY | 0.3217 | 1.1342 | 0.2886 | 0.8652 | 0.989 |
| KEGG_PROXIMAL_TUBULE_BICARBONATE_RECLAMATION | 0.4051 | 1.1366 | 0.303 | 0.9064 | 0.987 |
| KEGG_CELL_ADHESION_MOLECULES_CAMS | 0.3676 | 1.1375 | 0.3211 | 0.9567 | 0.987 |
| KEGG_GRAFT_VERSUS_HOST_DISEASE | 0.4943 | 1.1497 | 0.3297 | 0.9748 | 0.985 |
| KEGG_LEISHMANIA_INFECTION | 0.4096 | 1.1736 | 0.2964 | 0.9593 | 0.978 |
| KEGG_NEUROACTIVE_LIGAND_RECEPTOR_INTERACTION | 0.3191 | 1.1751 | 0.2351 | 1 | 0.978 |
| KEGG_RENIN_ANGIOTENSIN_SYSTEM | 0.4415 | 1.1863 | 0.2668 | 1 | 0.971 |
| KEGG_CYTOKINE_CYTOKINE_RECEPTOR_INTERACTION | 0.3542 | 1.2081 | 0.2448 | 1 | 0.963 |
| KEGG_ALPHA_LINOLENIC_ACID_METABOLISM | 0.4453 | 1.2217 | 0.2298 | 1 | 0.959 |
| KEGG_ALLOGRAFT_REJECTION | 0.5303 | 1.2439 | 0.2806 | 1 | 0.944 |
| KEGG_ALDOSTERONE_REGULATED_SODIUM_REABSORPTION | 0.4042 | 1.2632 | 0.175 | 1 | 0.934 |
| KEGG_ARACHIDONIC_ACID_METABOLISM | 0.4053 | 1.2789 | 0.171 | 1 | 0.918 |
| KEGG_AUTOIMMUNE_THYROID_DISEASE | 0.4715 | 1.3252 | 0.217 | 1 | 0.872 |

| Term | ES | NES | NP | FDR | FWER |
|---|---|---|---|---|---|
| KEGG_HEMATOPOIETIC_CELL_LINEAGE | 0.4655 | 1.3615 | 0.1673 | 1 | 0.844 |
| KEGG_TASTE_TRANSDUCTION | 0.5084 | 1.4813 | 0.0057 | 0.7741 | 0.69 |
| KEGG_TAURINE_AND_HYPOTAURINE_METABOLISM | 0.6345 | 1.5198 | 0.0517 | 0.7885 | 0.639 |
| KEGG_LINOLEIC_ACID_METABOLISM | 0.5449 | 1.5554 | 0.0419 | 0.8586 | 0.588 |
| KEGG_ASTHMA | 0.6458 | 1.568 | 0.088 | 1 | 0.567 |
| KEGG_INTESTINAL_IMMUNE_NETWORK_FOR_IGA_PRODUCTION | 0.6384 | 1.6303 | 0.0643 | 1 | 0.477 |

## Supplementary Figures

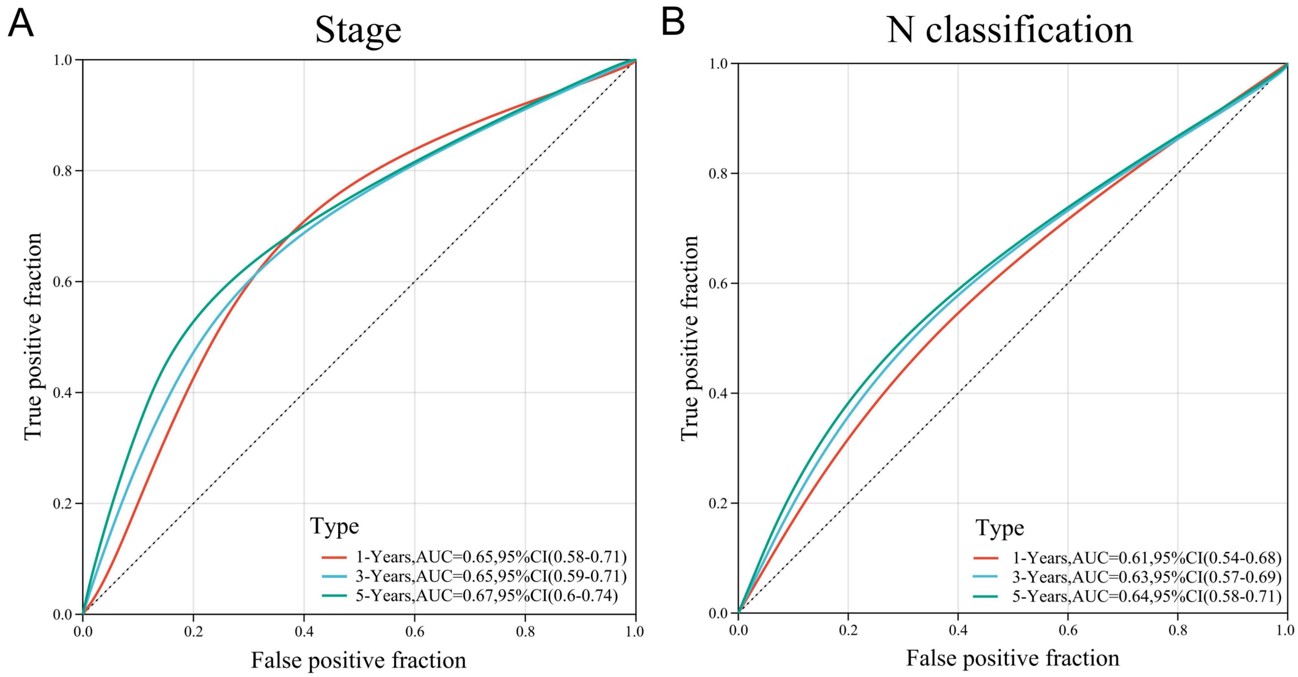


**Figure S1. ROCs.** A 1-year, 3-year, and 5-year ROC curves plotted based on (A) stage and (B) N stage.

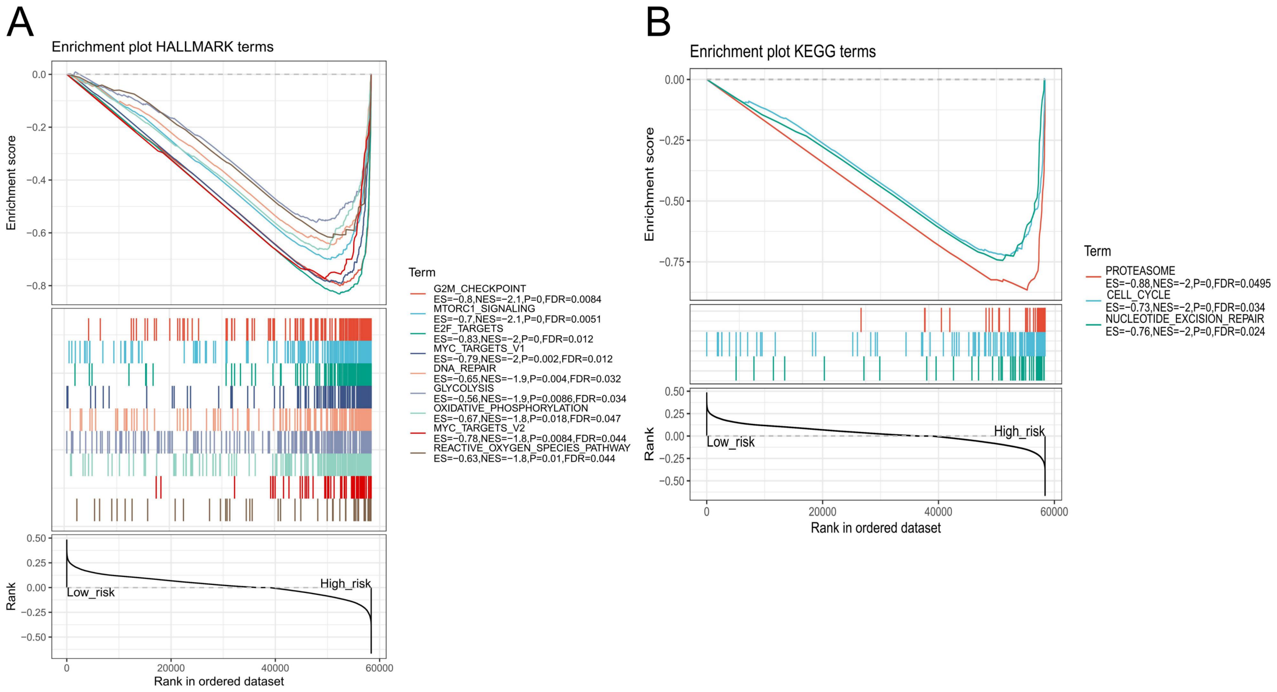


**Figure S2. GSEA analysis.** (A) Hallmark gene sets correlated with lncRNAs in the prognostic model: G2M checkpoint, mTORC1 signaling, E2F targets, MYC targets, DNA repair, glycolysis, oxidative phosphorylation, and reactive oxygen species pathway (P <0.05 and FDR < 0.05). (B) Involved regulated pathways: proteasome, cell cycle, and nucleotide excision repair (P <0.05 and FDR < 0.05).
